# Optimization of a formula to develop iron-dense novel composite complementary flour with a reduced phytate/minerals molar ratio from *dabi* teff-field pea-based blends using a D-optimal mixture design

**DOI:** 10.3389/fnut.2023.1244571

**Published:** 2023-10-11

**Authors:** Diriba Chewaka Tura, Tefera Belachew, Dessalegn Tamiru, Kalkidan Hassen Abate

**Affiliations:** ^1^Department of Nutrition and Dietetics, Institute of Health, Jimma University, Jimma, Ethiopia; ^2^Department of Human Nutrition, Wollega University, Nekemte, Ethiopia

**Keywords:** *dabi* teff, optimization, iron dense, phytate/minerals molar ratio, novel complementary food

## Abstract

Iron deficiency anemia is one of the major public health problems in children associated with the inadequate intake of bioavailable iron. Thus, this research was aimed at incorporating *dabi* teff, an underutilized/forgotten crop, into other pre-processed local food crops, *viz.*, germinated maize, roasted barley, roasted field pea, dehulled oats, and linseed, to develop optimized iron-dense novel composite complementary flour with a reduced phytate/minerals molar ratio. Nutrisurvey software was employed to define ranges, and they were constrained at 20–35% *dabi* teff, 0–30% field pea, and 5–20% maize, while the remaining were kept constant at 25% barley, 15% oats, and 5% linseed. Eleven experimental runs were generated from the six mixture components using Stat-Ease Design Expert^®^ software version 11, D-optimal. Inductively coupled plasma-optical emission spectrometry was used to determine micronutrients. ‘Scheffe’ regression was used to fit and test the model’s adequacy, and numerical multi-response optimization was performed using the Design Expert^®^ to identify the optimal points. *Dabi* teff had a significantly higher (*p* < 0.05) iron content at 86.5 mg/100 g, iron density at 24.53 mg/100 kcal, and calcium content at 123.59 mg/100 g. The new formulations had a significantly higher iron content (3.31–4.36 times), iron density (3.25–4.27 times), and calcium content (1.49–1.58 times), as compared to the control flour, and fulfilled FAO/WHO recommendations. The optimal formula was identified at 34.66% *dabi* teff, 25% barley, 15% oats, 15.34% field pea, 5% linseed, and 5% maize flour ratios, with response values at the overall optimization as 32.21 mg/100 g iron, 77.51 mg/100 g calcium, 2.59 mg/100 g zinc, 0.233 phytate/iron molar ratio (Ph:Fe), 0.067 phytate/calcium molar ratio (Ph:Ca), 3.43 phytate/zinc molar ratio (Ph:Zn), and 6.63 phytate by calcium to zinc molar ratio (Ph*Ca:Zn). Furthermore, it contained iron at a level that is 2.01 times higher than the standard and 4.44 times higher than the control, as well as an iron density of 8.47 mg/100 kcal, which was 4.39 times higher than that of the control. These findings showed that the optimized *dabi* teff-field pea based iron-dense novel composite complementary flour with enhanced bioavailability can be developed and used as a sustainable food-based strategy to combat iron deficiency anemia among children in less developed countries, such as Ethiopia.

## 1. Introduction

Micronutrient malnutrition is a serious, life-threatening public health problem in both developed and developing countries (1). Its deleterious effect is more significant in children under 5 years, where more than 50% of the children worldwide experience one or more key micronutrient deficiencies, and it often co-exists with infectious diseases (2). The adverse health consequences of micronutrient deficiency in children include impaired physical and cognitive development, diminished learning and earning capacity, and reduced immune competency, which may be difficult to reverse by nutrition interventions (3, 4).

The most often deficient micronutrients in developing countries include iron, zinc, calcium, vitamin A, and iodine, among others, and their deficiency is mostly superimposed by protein-energy malnutrition, referred to as ‘hidden hunger’ (5). Iron deficiency is the most prevalent micronutrient deficiency affecting approximately two billion people around the world. Anemia is a disease with multiple causes, and it includes both nutritional anemia (iron and vitamin deficiencies) and non-nutritional anemia (infection and blood loss). Among nutritional anemia, iron deficiency anemia is the most common in children worldwide (6). Global estimates show that the highest prevalence of anemia was 47.4% among under-five children, with the prevalence rising to 64.6% in Africa (7). According to the Ethiopian Public Health Institute (EPHI) and ICF (8) survey, 57% of children under 5 years were anemic.

Micronutrient deficiency-related growth impairments usually occur during the complementary feeding period due to the high demand for varied micronutrients at this age (9). Hallberg and Rossander (10) reported that micronutrient malnutrition, especially anemia, is more prevalent in children between 6 and 24 months of age than at any other time in the life cycle, and the major causes are inadequate dietary intake of bioavailable iron, malaria, and parasitic infections. For example, for 9–11-month-old children, approximately 97% of iron, 86% of zinc, 72% of calcium, 76% of magnesium, 81% of phosphorus, and 73% of sodium should come from complementary food, assuming average breast milk intake (11–13).

Zinc is a vital micronutrient, and its deficiency leads to stunted growth and infectious diseases. Globally, it was estimated that zinc deficiency is responsible for approximately 16% of lower respiratory tract infections, 18% of malaria, and 10% of diarrhea cases (14). Calcium serves as a component of bones and teeth, a regulator of nerve and muscle functions, and a regulator of blood clotting and many other functions, and its deficiency usually leads to stunted growth in children and bone loss or osteoporosis in adults (15).

Traditional complementary foods in developing countries, including Ethiopia, are predominantly cereal-based; they are often monotonous and are characterized by their low nutritive value, low micronutrient density, and high anti-nutritional factors, such as phytates, tannins and other phenolic compounds, and fibers, which jeopardize the nutrient bioavailability of the products (16, 17). Thus, the very high demand for micronutrients is unlikely to be met by infants and young children using traditional complementary foods. Animal-source foods, such as meat, liver, and fish, can provide for the demand as they contain higher bioavailable minerals. However, these products are often expensive and unaffordable for low-income households, particularly the rural poor with additional religious concerns (17). Consequently, in developing countries, it was suggested that extending child breastfeeding beyond 2 years is the first line of defense against key micronutrient deficiencies, such as iron deficiency anemia, although the iron content of human milk is relatively low (12).

Micronutrient supplementation and fortification were proposed as effective strategies to improve mineral bioavailability and combat micronutrient deficiencies (18), although they posed limitations in developing countries due to reduced accessibility to such programs. In Ethiopia, efforts were being made to implement a multi-sectorial plan of high-impact nutrition-specific and nutrition-sensitive intervention, such as the Multi-Sectoral Nutrition Coordination and Integration Guideline (2017), the Nutrition Sensitive Agriculture Strategy (2016–2020), and the School Feeding Program (2019), to end child under-nutrition, including eradicating micronutrient deficiencies by 2030 (19, 20). However, the country is still experiencing one of the worst situations where micronutrient deficiencies, including iron, zinc, and vitamin A deficiencies, are unacceptably high among infants and young children. This calls for searching for other promising alternatives, such as exploring locally available underutilized/forgotten nutritious food crops, to develop optimized micronutrient-dense composite complementary flour, which has been suggested to be the best food-based approach (21, 22).

The food-based or dietary change approach is a combination of nutrient-rich food production and consumption that is oriented toward children, pregnant women, and lactating women. The approach provides several micronutrients at once, is affordable and sustainable, and can be adapted to different cultural and dietary traditions, as well as locally feasible strategies (21, 22). It focuses on the combination of diversified foods that can meet the nutrient requirements of the target group and is based on the fact that people eat foods, not nutrients or supplements (23, 24). The FAO/WHO (25) codex alimentarius on complementary feeding guidelines describes that mixtures of cereals, legumes, and pulses/oilseed can constitute an appropriate source of nutrients and energy, essential fatty acids, and limiting amino acids; they have many functional and health benefits, as well as improved organoleptic characteristics. Enzymatic processing, such as germination and fermentation, or non-enzymatic processing, such as roasting, soaking, maceration, and dehulling, are among the most commonly known techniques for reducing the deleterious effects of anti-nutrients and increasing the nutrient density and bioavailability of key micronutrients (23).

The crucial effects of roasting crops are that it reduces microorganisms, destroys insects, and inactivates trypsin enzyme inhibitors, thus improving protein digestibility, amino acids bioavailability, and keeping qualities; additionally, it enhances the flavor and taste of food products through starch dextrinization (25).

Germination is a bioprocess technology that might, in particular, increase protein digestibility and enhance the absorption and bioavailability of micronutrients, including vitamins such as riboflavin (B_2_), pyridoxine (B_6_), vitamin C, vitamin E, and antioxidants (21). Additionally, germination helps in the degradation of the phytate-mineral complex and polyphenol-protein complex and also decreases condensed tannin, thus facilitating mineral and protein absorption. Furthermore, the enzyme α-amylase synthesized during germination hydrolyzes starch to dextrin (dextrinization) and maltose (referred to as the amylase-rich food technology) and reduces its water-holding capacity, thereby reducing the viscosity of thick cereal-legume porridges and enhancing or maintaining their energy and nutrient densities for infants and young children who have limited gastric capacity (21, 25).

Maceration is a hydrothermal processing technique that involves hydration and heating; it enables the release of micronutrients from the plant matrix and improves the availability of food nutrients by destroying (leaching) anti-nutritional compounds. This wet-heat treatment of foods has been attributed to the increased solubility of some minerals, such as iron, calcium, and magnesium, which causes the leaching of the micronutrient into the processing water, thereby leading to the bulk micronutrient losses over long periods of maceration. Thus, short-time maceration is recommended to avoid such bulk losses of key micronutrients (26). The sub-Saharan African region is endowed with a rich diversity of ‘orphan crops,’ which have improved nutritional and medicinal values and are also income generation sources (27). Little or no attention has been paid to such crops in terms of research and development and policy framework, which could promote their extensive agricultural production, industrial utilization, as well as home consumption. *Dabi* teff (*Eragrostis teff*), a farmer-variety teff, is an underutilized/marginalized or forgotten food crop grown in Ethiopia (Oromia region) that could be utilized to combat micronutrient malnutrition.

*Dabi* teff is the ‘afaan oromoo’ language name for an early maturing variety of dark red teff, due to which the crop is harvested twice within one rainy season (during early rainfall called “*daabii* gannoo” and during late rainfall called “*daabii* birraa”). Early maturation and low rainfall requirements make *dabi* teff a unique variety of teff amenable to climate-smart agriculture. Farmers in Wollega and Illuababor, western Ethiopia, cultivate *dabi* teff either for its grain seeds or its straw. The contribution of *dabi* teff grains to household food and nutrition security is considerable when viewed through a food security lens, while the straw is composed of fine stems used for plastering (finishing) mud hut walls during house construction and rated higher among others for animal fodder, especially oxen feeding (personal experience). Red teff varieties, including *dabi* teff grown in Ethiopia, are lower in price than the white varieties and have been implicated in the low incidence of anemia, which is presumed to be due to the red teff grain’s high iron content (28).

There are many social beliefs regarding the nutritional and health benefits of *dabi* teff among consumers; for example, the consumption of the different food forms of *dabi* teff products, such as *kita/maxinoo* (unleavened bread), *muk/mooqa* (gruel), *mooqa manyee,* and *cafaqoo* (the favorite cultural dishes), increases blood volume “*dhiiga dabalaa*,” boosts energy/strength “*humna dabalaa,*” and repairs/strengthens the backbone and fractured bones “*dugda jabeessa,*” which could be implicated for its high calcium content. *Dabi* teff is prized as a medicinal food among rural elderly people, in particular, and consumers, in general (based on personal communication).

Based on these social beliefs and personal long-time observations, we conducted a preliminary study on some selected micronutrient contents of *dabi* teff and found that it contains a considerable amount of iron at 122.66 mg/100 g, calcium at 190 mg/100 g, and zinc at 1.8 mg/100 g (preliminary findings by the researcher), which created a pivotal springboard to carry out intensive research work.

Despite the social beliefs and the preliminary findings, to date, the potential of incorporating *dabi* teff into complementary flour formulations to combat micronutrient malnutrition in infants and younger children has not been yet examined. Thus, this research was aimed at incorporating *dabi* teff into pre-processed local food crops, *viz.*, germinated maize, roasted barley, roasted field pea, dehulled oats, and minimally cooked linseed, to optimize a formula; this formula was aimed at developing novel iron-dense composite complementary flour from *dabi* teff-field pea-based blends, identifying the optimal formula (sweet spot) with enhanced bioavailability using the D-optimal mixture design, and suggesting the promising potential of the *dabi* teff-field pea-based novel composite complementary food to combat iron deficiency anemia among children in Ethiopia and Africa in general.

## 2. Materials and methods

### 2.1. Food crop sample collection

Food crops, *viz.*, *dabi* teff (*Eragrostis teff (Zucc.)* farmer variety), maize (*Zea mays L.*), barley (*Hordeum vulgare*), white field pea (*Pisum sativum*), oats *(Avena sativa)*, and linseed (*Linum usitatissimum*), were purchased from an open market in Nedjo town, Oromia, Ethiopia, which is located 575 km to the west of Addis Ababa, where Nedjo district is a potential *dabi* teff growers. The availability of these crops in the area was verified by an inspection survey of the Nedjo market, as well as by asking mothers about the traditional usage of the crops as complementary foods and their affordability. Literature-based nutrient content of each crop was considered during the selection process, which was later verified through micro-composition analysis (Table 1), and the crops were wisely selected to add one or more essential nutrients to their mixture for complementation. Apparently uninfected samples were purchased from the center and corners of the market on two different market days to assure representativeness. Each piece of the six crops purchased from the different corners was packed separately in polyethylene bags and transported to the Ethiopian Institute Agricultural Research (Food Science and Nutrition laboratory) and mixed using a laboratory mixer (Hot Sale DZ-10 L laboratory mixer, China) to obtain homogeneous 3 kg of composite crop samples each. All laboratory analyses of the micronutrients and the anti-nutritional factors were conducted in the stated laboratory, which was certified by the International Organization for Standardization (ISO) (ISO-17025:2017) by the International Laboratory Accreditation Cooperation (ILAC).

**Table 1:** Dietary mineral contents of the individual components processed flour.

Individual flour components	Micronutrient contents (mg/100 g)						
	Iron	Calcium	Zinc	Magnesium	Potassium	Phosphorous	Sodium
Dabi teff	86.50 ± 0.42^a^	123.59 ± 1.02^a^	1.94 ± 0.04^a^	142.48 ± 0.39^a^	297.97 ± 3.97^a^	237.14 ± 1.43^a^	4.90 ± 0.31^a^
Roasted barley	5.00 ± 0.06^b^	32.49 ± 0.76^b^	2.46 ± 0.03^b^	87.48 ± 1.06^b^	355.45 ± 6.11^b^	279.09 ± 2.13^b^	3.94 ± 0.40^a^
Dehulled oats	4.37 ± 0.07^bc^	26.33 ± 0.59^c^	2.28 ± 0.03^c^	78.87 ± 0.44^c^	319.04 ± 6.74^c^	261.23 ± 2.32^c^	1.57 ± 0.51^b^
Roasted field pea	7.05 ± 0.19^d^	59.99 ± 0.61^d^	2.28 ± 0.02^cd^	89.54 ± 1.44^bd^	720.14 ± 4.41^d^	227.58 ± 7.82^a^	1.69 ± 0.77^bc^
Cooked linseed	47.13 ± 0.33^e^	28.83 ± 0.23^e^	6.63 ± 0.10^e^	279.86 ± 2.23^e^	563.28 ± 6.02^e^	488.97 ± 0.78^b^	7.91 ± 0.51^d^
Germinated maize	1.12 ± 0.02^f^	15.78 ± 0.45^f^	1.56 ± 0.06^f^	80.91 ± 0.47c^f^	260.20 ± 1.67^f^	236.48 ± 4.07^a^	3.73 ± 0.47^a^

Values are means ± Standard deviation of the triplicate determinations. Values in the same column followed by different superscripts are significantly different at *p* < 0.05.

### 2.2. Samples processing

**Figure 1:**
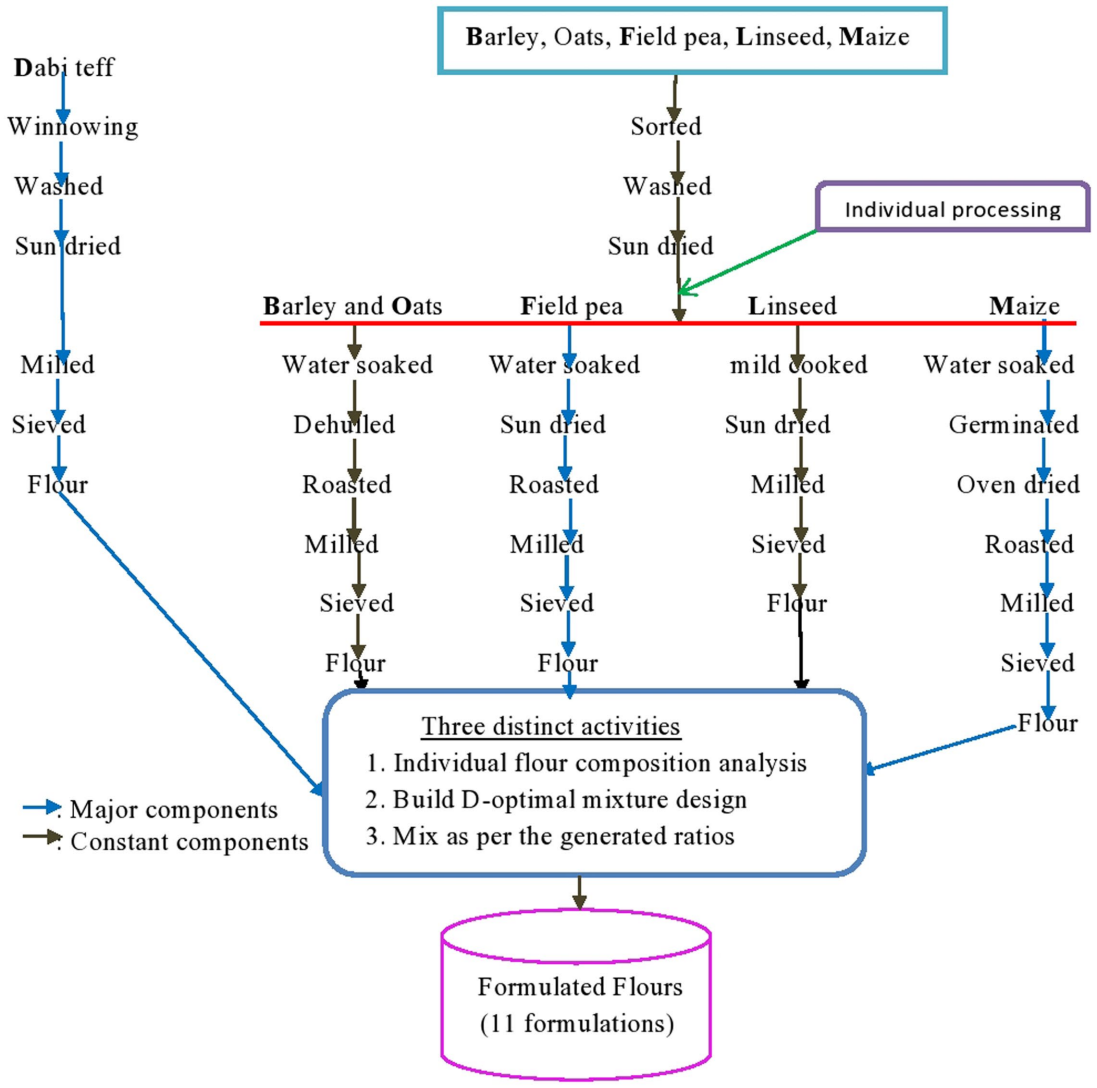
Process flowchart diagram of the crop samples and formulations.

It is not only the proper selection of nutritionally promising crops but also the processing techniques applied that determine the quality of the final product. Accordingly, the collected crop samples underwent various controlled processing techniques. In brief, *dabi* teff was manually cleaned by winnowing to remove chaffs, straw, dust, and other extraneous materials and washed with tap water and sundried, while the other cereals (maize, barley, and oats) and legumes (field pea and linseed) were sorted out from sands, sticks, stones, and defective seeds, washed later and sundried for 2 days, and were ready for further individual processing (Figure 1). Due to the small size of *dabi* teff seeds, it was made into whole-seed milled flour, which could be the reason for the higher nutrient contents of the crop. Barley and oats samples were soaked in clean tap water for 2 h; the water used for soaking was drained off, the crops were immediately decorticated (while the seeds were still wet) using a wooden decorticator, and the hulls were removed by winnowing.

Germination of maize seeds was adapted from the method previously described by Rasane et al. (29), with little modification. Briefly, the maize grains were soaked in water (1.3 w/v) for 3 h to achieve hydration, drained, spread on a clean jute sack placed on a wooden platform, and covered with another jute sack for germination at room temperature (25°C ± 2°C) for 72 h; water was sprayed every 12 h to keep them humid (60% relative humidity). The time span of 72 h was chosen because Rasane et al. (29) reported that the maximum amylase activity was obtained at 72 h of germination time. After 72 h, the germinated seeds were rinsed, drained for 10 min, transferred to aluminum trays, and dried in an air oven at 40°C for 5 h to terminate the germination process. The dried germinated sample was further roasted at 120 ± 5°C for 10 min and allowed to cool.

The crop samples, *viz.*, barley, oats, field peas, and germinated maize, were minimally roasted in an oven at 120°C for 20 min until they were light brown colored, and then they cooled to room temperature (25 ± 2°C), as described by Rasane et al. (29). The roasting process was carefully controlled to prevent over-roasting, as well as to prevent the formation of undesirable Maillard reactions that may lead to protein quality damage. The linseed sample was minimally cooked for 5 min at 90°C (30) with a small amount of water to condition the seeds to release oil from the oil cells; they were later sundried without draining the water used for cooking.

### 2.3. Flour preparation and handling

All six processed samples were milled into flours using a standard miller (Cyclotec 1,093 sample mill, Foss Analytical, Sweden) to obtain smooth and consistent particle sizes and sieved through a 0.5 mm mesh sieve size. The flours were then packed in air-tight high-density polyethylene bags (31), separately coded, and stored safely at room temperature until formulation.

### 2.4. Experimental design

#### 2.4.1. Flour formulation

Approximately 100 g of flour from each of the six components was taken separately before generating the formulation to determine the mineral contents of each sample (Table 1).

In the current study, Stat-Ease Design Expert^®^ software version 11 (D-optimal, Minneapolis, USA, 2018) was used to generate the formulation design matrix (Table 2). The range of each component in the formulation was defined based on three considerations: first, targeting to attain the FAO/WHO (25) recommendations; second, aligning with the Ethiopian complementary feeding guidelines (32); and third, accounting for the micronutrients of individual components.

**Table 2:** D-optimal mixture design matrix, formula code, mixture ratio, control and individual flours and constraints with their limits.

Std. order	Design ID	Run order	Mixture components ratio (%)						Limits of mixture components			
Standard	ID	Formulated code	X1	X2	X3	X4	X5	X6	Total	Constraints	Lower	Upper
2	0	F1	27.5	25.0	15.0	15.0	5.0	12.5	All	X1	20	35
7	10	F2	26.123	25.0	15.0	23.877	5.0	5.0	100	X2	25	25
10	4	F3	30.457	25.0	15.0	19.543	5.0	5.0		X3	15	15
11	5	F4	20.000	25.0	15.0	29.988	5.0	5.012		X4	0	30
1	2	F5	28.793	25.0	15.0	6.207	5.0	20.0		X5	5	5
9	3	F6	35.000	25.0	15.0	11.884	5.0	8.116		X6	5	20
8	1	F7	21.705	25.0	15.0	13.295	5.0	20.0				
4	7	F8	34.385	25.0	15.0	6.476	5.0	14.139				
5	8	F9	20.000	25.0	15.0	19.54	5.0	15.46				
3	6	F10	22.735	25.0	15.0	22.515	5.0	9.75				
6	9	F11	29.604	25.0	15.0	10.076	5.0	15.32				
		Control	0	80	0	0	0	20				
		Dabi teff	100	0	0	0	0	0				
		Barley	0	100	0	0	0	0				
		Oats	0	0	100	0	0	0				
		Field pea	0	0	0	100	0	0				
		Linseed	0	0	0	0	100	0				
		Germinated maize	0	0	0	0	0	100				
		Optimized flour	34.66	25	15	15.34	5	5				

Std., Standard; ID, design identity; x_1_, Dabi Teff; x_2_, Barley; x_3_, Oats; x_4_, Field Pea; x_5_, Lin Seed; x_6_, Germinated Maize; F1-F11, formula code for each formulation.

The micronutrient content analysis results of individual components (Table 1) were recorded (customized) into Nutrisurvey analysis (Version 2007) (Table 3). By estimating the amount of a meal to be consumed by 1-3-year-old children to be 75 g (solid portion) per meal and adjusting for the required number of meals per day, several trials (iterations) were made to define the percentage ranges (Table 2) of major components (*dabi* teff, field pea, and germinated maize); this was done by entering the range-related amounts into the software, which were combined with the constant components, and the output was examined. The final generated output showed the percentage of fulfillment by the meal mixture compared to the daily recommended micronutrient intake (RNI) for 1-3-year-old children by the Pan American Health Organization and World Health Organization (PAHO/WHO) (11). The fulfillment percentages for various nutrients were as follows: 84–152%, 90–188%, 107–195%, 41–61%, 612–1,045%, 133–225%, and 229–400% for energy, protein, carbohydrate, fat, iron, zinc, and magnesium, respectively (Table 3) which corresponded to 20–35% of *dabi* teff, 0–30% of field pea, 5–20% of germinated maize, 25% of barley, 15% of oats, and 5% of linseed meal mixture, and these were used as constraints for generating the formulation matrix (Table 2). The conventional flour (a commonly used cereal-based complementary food in the sample collection area) was used as control, constructed from 80% barley flour and 20% ungerminated maize flour (personal communication or information collected from some skilled caretakers/mothers assisted by primary health extension works in the village).

**Table 3:** Nutrisurvey analysis of the food records (customized) to estimate the ranges of each component for formulations.

		Calculated energy and nutrient values of the formulated meal by nutrisurvey								
Components	Proportion (%) (lower-upper)	Amount eaten range/day (g)	Energy range (kcal)	CHO range (g)	Protein range (g)	Fat range (g)	Iron range (mg)	Calcium range(mg)	Zinc range (mg)	Magnesium range (mg)
Dabi teff	20–35	45–79	158.7–278.7	30.9–54.2						
Roasted barley	25	56	212.9	43.5						
Dehulled oats	15	34	129.5	23.8						
Roasted field pea	0–30	0–68	0–238.3	0–41.1						
Cooked linseed	5	12	62.4	3.4						
Germinated maize	5–20	11–45	41.4–169.5	8.3–33.9						
Analyzed value (range) for the formulated meal using our components			604.9–1091.5*	109.9-199.9 (72.7–73%)	19–39.8 (13–15%)*	10-14.8 (15-12%)	49–83.6*	87.8-176.1	4-6.7*	183-320.3
Recommended value for 1–3 years old child as generated by the ‘Nutrisurvey’ software			718.3	102.5 (>55%)	21.2 (12%)	24.4 (<30%)	8	600	3	80
Percentage fulfillment from the formulated meal (range) (lower-upper) %			84–152*	107–195*	90–188*	41–61	612–1,045*	15–29	133–225*	229–400%

*Showing the blended complementary flours would be promising to attain the FAO/WHO recommendations.

The D-optimal mixture design was run to generate the formulation matrix using the range defined and later used to optimize the formulation. The six components were constrained to generate a total of 16 experimental runs with 6 central points and 5 replicates to provide 11 experimental runs with an estimate of pure error and 5 lack-of-fit points. The amount in grams of the individual components generated in each formulation (experimental run) was carefully weighed on a digital balance gravimetrically and put together, after which it was thoroughly mixed using an electrical blender for 3 min at 200 rpm to homogenize the mixture. Finally, it was packed and sealed in high-density polyethylene bags and stored in a refrigerator at 4°C until analysis.

#### 2.4.2. Dietary mineral analysis of the individual components and formulations

Analysis of aqueous solutions by inductively coupled plasma-optical emission spectrometry (ICP-OES) was employed for the simultaneous determination of the dietary mineral contents (Fe, Zn, Ca, Mg, K, P, and Na) of the individual components. This method was also used for the analysis of the formulated composite complementary flours with a radial plasma observation method (33). All the measurements were performed using a sector arcos optical emission spectrometer optimized with a small volume and 32 linear charge-coupled device (CCD) detectors in the wavelength range between 130 and 770 nm, and they were simultaneously analyzed. A nebulizer was used to introduce the sample solution into the argon gas field plasma, and the energy excitement was preceded in the single spectra. The energy was expressed in terms of intensity, which was directly proportional to concentration; the concentration was calculated on the linear graph of the standard concentration and the corresponding intensities. All the measuring conditions, such as plasma power, gas flow, torch positions, and measuring time, were configured for concentration measurements. According to the standard, the calibration and standardization of the spectra method were performed, and standardization was performed daily. It is a quick procedure for correcting measuring intensities so that the correct concentration is obtained using the original calibration curve.

#### 2.4.3. Determination of anti-nutritional factors (non-nutrient contents)

##### 2.4.3.1. Phytate content determination

Latta’s and Eskin’s (34) method was used to determine the phytate content after sample extraction with 10 mL of 0.2 N HCl for 1 h, centrifuged (Sigma 2-16KC, UK), and the clear supernatant sample extract (3 mL) was reacted with 2 mL of Wade reagent (0.03% FeCl3.6H2O and 0.3% sulfosalicylic acid in distilled water) and vortexed mixed for 5 s. The absorbance of the sample was measured at 500 nm using a UV–visible spectrophotometer (V-630, Jasco, USA). The phytate concentration was calculated from the difference between the absorbance of the blank (3 mL of 0.2 N HCl + 2 mL of Wade reagent) and that of the assayed sample. It was calculated using a phytic acid standard curve prepared under the same condition, and the result was expressed as mg/100 g.

##### 2.4.3.2. Condensed tannin content determination

Traditionally, tannins have been classified into condensed and hydrolyzable tannins, based on their hydrolytic properties in hot water or their response to the tannase enzyme. In the present study, condensed tannin (proanthocyanidins) was analyzed by the Vanillin–HCl method of Price et al. (35). The sample was milled until it could pass through a ≤ 750 μm sieve. Approximately 200 mg of the sample was weighed into a screw-capped test tube and extracted with 10 mL methanol by vortex-mixing for 20 min. Then, it was centrifuged at 3,000 rpm for 10 min. The Vanillin (5 mL) reagent was mixed with 1 mL of the sample extract at 1 min intervals into a one-test sample set, and for the blank (1 mL), only 0.5 mL of concentrated HCl was added in methanol 4% at 1 min intervals. The supernatant was used for the analysis after warming it along with the Vanillin-HCl reagent in a water bath at 30°C. One milliliter of the sample extract was taken in duplicate for each sample (one for mixing with the Vanillin reagent and the other as a blank) to be deducted from sample absorbance. Then, it was immediately incubated in a water bath at 30°C for 20 min. After 20 min, the absorbance was immediately measured/read on a spectrophotometer at 500 nm in 1 min intervals, as per the sequence used for mixing. The sample absorbance was deducted from the blank, and the value was estimated from the catechin equivalent (CE) standard curve. A standard curve absorbance (y) against the catechin concentration (x) was prepared from the catechin. Standard solution readings and the slope were computed, and the tannin concentration was determined from the equation of the curve and expressed as mg/100 g.


$$CE=CC*\frac{VM}{VE*Wt}$$

where CE = catechin equivalent; CC = catechin concentration (mg/mL); VM = volume made up (mL); VE = volume of extracts, and Wt = weight of the sample.


$$\text{Tannin in mg/g = }\frac{\left(As-\mathrm{Ab}\right)-\mathrm{Intercept}}{\mathrm{Slope}\times D\times W}$$

where As = sample absorbance; Ab = blank absorbance; D = density of the solution (0.791 g/mL); and W = weight of the sample in gram.

#### 2.4.4. Phytate/minerals molar ratios (*in-vitro* minerals bioavailability)

The phytate/minerals molar ratio for each formulation for iron, calcium, and zinc was computed using the method of Norhaizan and Nor Faizadatul (36). The amount of phytate and minerals in each formulation was divided by their respective atomic weight (phytate: 660 g/mol; Fe: 56 g/mol; Zn: 65 g/mol; and Ca: 40 g/mol), and the phytate/mineral molar ratio was obtained by dividing the phytate mole with the mole of the respective minerals. The molar ratios found were compared with the acceptable critical limits (25), including the phytate/calcium molar ratio (Ph:Ca) (< 0.24), the phytate/iron molar ratio (Ph:Fe) (< 1), the phytate/zinc molar ratio (Ph:Zn) (< 15), and the Ph*Ca:Zn molar ratio (< 200), which are used as proxy indicators of good mineral bioavailability or uncompromised absorption.

#### 2.4.5. Determination of minerals density of the optimized formula

The mineral density of the optimized flour was calculated by dividing the mineral content at the optimal condition by its corresponding optimal energy value expressed as mg/100 kcal.

### 2.5. Statistical analysis and model evaluation

All the laboratory analysis results of the 11 formulations (Table 2) were subjected to Scheffe’s polynomial multiple regression analysis using Stat-Ease Design Expert^®^ software version 11 (D-optimal mixtures design). The mixture components were considered model terms, and the mixture regression was designated as the model fitting. Linear, quadratic, cubic, and special cubic models and interactive effects of the independent variables were fitted for the evaluation of dietary minerals, phytate, tannin, and phytate/mineral molar ratio (Table 4). Analysis of variance (ANOVA) of the design expert was performed to develop models and determine the goodness of fit (significance) of the models developed. Linear and polynomial regression models were judged (verified) to be adequate and significant using F-statistic at a probability (P) of 0.05, 0.01, and 0.001 and the coefficient of determination *R*^2^. *R*^2^% is the percentage variation of the response (dependent) variable that can be explained by its linear relationship with the independent variable, and it measures the degree of fitness of a regression model. The closer the *R*^2^ value is to unity (1), the better. The closer *R*^2^_adj_ is to *R*^2^_pred_ (with a difference of less than 0.2), the better is the model fit. An adequate precision value greater than 4 indicates satisfactory fitting of the model. The *R*^2^% value should be at least 80% to have a good regression model fit (37). Normality and constant variance assumptions of the error terms were checked to determine whether a model meets the assumptions of the analysis. Furthermore, datasets of the dietary minerals and anti-nutrient contents of the individual and formulated flours were statistically analyzed using a one-way ANOVA of SPSS (IBM version 24, Chicago, USA) to declare statistically significant differences between the formulated flours, control, and the Cerifam^®^ faffa flour (a popular commercial complementary/weaning flour in Ethiopia). All the data collected were in triplicate. Levene’s test was conducted to check the equal variance assumptions (*p* > 0.05, should be non-significant). Tukey’s honestly significant difference (HSD) post-hoc test was conducted for pairwise multiple comparisons (mean difference separation test), and significant differences were declared at a value of *p* of <0.05.

**Table 4:** Models fitted for responses and statistical outputs showing significance of regression models and model adequacy.

	Mineral contents			Phytate/Minerals molar ratio			
Model’s *P*-value (model terms)	Iron	Calcium	Zinc	Ph:Fe	Ph:Zn	Ph:Ca	Ph^*^Ca:Zn
Linear (x_1_,x_4_,x_6_)	0.0001#***	0.1959#^	0.0164#*	0.0001#***	0.0269#*	0.101	0.0240#*
Quadratic (x_1_^2^, x_4_^2^, x_6_^2^)	0.2371	0.4114	0.4476	0.8767	0.4914	0.156#^	0.6042
Special Cubic	0.4765	0.4444	0.1574	0.6340	0.6445	0.837	0.7531
Cubic	0.2183	0.9156	0.8113	0.3527	0.5676	0.648	0.3427
Sp. Quartic vs. Quadratic	0.5088	0.8675	0.2753	0.0623	0.3208	0.455	0.3105
Interaction terms							
x_1_x_4_	-	-	-	-	-	0.036	-
x_1_x_6_	-	-	-	-	-	0.122	-
x_4_x_6_	-	-	-	-	-	0.502	-
x_1_x_4_x_6_	-	-	-	-	-	-	-
F-statistics (Anova)							
Model *F*-value	59.3	2.01	7.18	63.43	5.88	3.66	6.16
Lack-of-Fit	PE0	PE0	PE0	PE0	PE0	PE0	PE0
*R* ^2^	0.9368	0.3347	0.6423	0.9407	0.5949	0.786	0.6064
*R*^2^%	93.68	33.47	64.23	94.07	59.49	78.56	60.64
ADP	18.63	3.73	6.94	19.25	6.35	5.4	6.46
Model	0.0001*	0.1959^	0.0164*	0.0001*	0.0269*	0.09^	0.0240*

Sp., special; vs, versus; PEO, pure error zero; *R*^2^%, coefficient of determination; ADP, adequate precision; Ph:Fe, phytate to iron; Ph:Zn, phytate to Zinc; Ph:Ca, phytate to calcium; #, suggested; #*, #** and #***, model is suggested and significant at *p* < 0.05, at *p* < 0.01 and at *p* < 0.001, respectively; #^, suggested and not significant; * and ^ model is significant and not significant respectively; x_1_, dabi teff; x_4_, field pea; x_6_, germinated maize.

### 2.6. Variable optimization and validation

After regression analysis of the experimental data, numerical and graphical optimization techniques were employed to identify the optimum formula (sweet spot) using the design expert (D-optimal mixture design). Simultaneous numerical values of independent variables and multi-response optimization were performed by setting the desired goals for each independent and dependent (response) variable. In brief, the major components used, *dabi* teff flour, field pea flour, and germinated maize flour, were set in the range, and the responses were set to maximize iron, calcium, and zinc and minimize phytate and phytate/minerals molar ratios (Table 5). In addition to goal setting, the relative importance of each dependent variable in the overall optimization solution was set to be ‘+++++’ for iron, calcium, Zn, and phytate/minerals molar ratios, while the remaining were kept at default, which is ‘+++’. These settings are important because the overall desirability function value of an optimization process is majorly ‘Goal or criteria setting-dependent’.

**Table 5:** Goals set, relative importance of each variable, and the optimal values at optimal condition identified.

Name	Goal	Lower limit	Upper limit	Importance	Optimum value
Independent variables					
A:Dabi teff Flour	In range	20	35	3	34.66
D:Field pea Flour	In range	0	30	3	15.34
F:Maize Flour	In range	5	20	3	5
Barley flour	Constant	25	25	3	25
Oats flour	Constant	15	15	3	15
Linseed flour	Constant	5	5	3	5
Dependent variables					
Iron (mg/100 g)	maximize	24.007	31.5801	5	32.21
Calcium (mg/100 g)	maximize	73.4631	78.1767	5	77.51
Zink (mg/100 g)	maximize	2.326	2.612	5	2.59
Magnesium (mg/100 g)	maximize	113.899	124.223	3	118.64
Potassium (mg/100 g)	maximize	364.768	497.482	3	439.33
Phosphorous (mg/100 g)	maximize	281.028	308.538	3	287.99
Sodium (mg/100 g)	maximize	3.455	6.246	3	4.44
Phytate (mg/100 g)	minimize	85.2	104.12	5	86.01
Ph:Fe	minimize	0.232	0.344	5	0.233
Ph:Zn	minimize	3.356	4.18	5	3.43
Ph:Ca	minimize	0.067	0.085	5	0.067
Ph*Ca:Zn	minimize	6.457	7.943	5	6.63

Graphical optimization was carried out by the superimposition of contour plots for all the responses with respect to the component ratios. To confirm the validity of the models, a laboratory experiment was conducted on the component ratios identified at the optimal formula, and the obtained laboratory results were then compared with the optimal values of the responses at the optimal condition and with the values of the control flour. They were finally reported as the formula with the optimal nutritional profile of all the possible combinations or solutions. A one-way ANOVA using SPSS (IBM version 24, Chicago, USA) was conducted to compare the means and declare the statistically significant differences between the predicted response values, the laboratory analysis (validation value), and the control value at a value of *p* of <0.05. Furthermore, two sample *t*-tests were conducted to compare the predicted optimal response values with the validation (laboratory analysis) values to confirm the validity and adequacy of the model equations to predict the optimal response values at a value of *p* of <0.05.

## 3. Results

### 3.1. Dietary mineral contents of the individual processed flours

In the current study, seven different dietary minerals were studied for their contents in the individual components as well as in the formulated composite flours. Iron, calcium, and zinc were emphasized more than other minerals in the formulated composite flours because these micronutrients are limited in the diets of infants and young children and are strongly associated with children’s physical growth, mental development, and cognitive development, and deficiency diseases related to them are most common (12). In this study, the mean and standard deviations of the mineral contents of the individual components used are presented in Table 1. A significant difference (*p* < 0.05) was observed in the mean values of mineral contents among the components, ranging between 1.12 and 86.5 mg/100 g for iron, 15.78 and 123.59 mg/100 g for calcium, 1.56 and 6.63 mg/100 g for zinc, 78.87 and 279.86 mg/100 g for magnesium, 260.2 and 720.14 mg/100 g for potassium, 227.58 and 488.97 mg/100 g for phosphorous, and 1.57 and 7.91 mg/100 g for sodium (Table 1). *Dabi* teff flour showed the highest (*p* < 0.05) amount of iron and calcium contents at 86.5 mg/100 g and 123.59 mg/100 g, respectively, compared to the other components (Table 1). The second-highest iron content was recorded for linseed flour at 47.13 mg/100 g, whereas the remaining components contained significantly lower iron, with germinated maize having the least at 1.12 mg/100 g.

The calcium content of *dabi* teff was significantly higher (*p* < 0.05) at 123.59 mg/100 g than that of the others, followed by roasted field pea flour at 59.99 mg/100 g. Linseed flour contained a significantly higher (*p* < 0.05) amount of zinc content at 6.63 mg/100 g, followed by roasted barley flour at 2.46 mg/100 g, while *dabi* teff flour showed a lower zinc content at 1.94 mg/100 g. Similarly, linseed contained a significantly higher (p < 0.05) magnesium content at 279.86 mg/100 g, followed by *dabi* teff flour at 142.48 mg/100 g.

### 3.2. Anti-nutritional factors (phytate and tannin) of the individual processed flours

The mean phytate contents of the component were significantly different at a value of *p* of <0.05 and ranged between 80.6 mg/100 g and 158.9 mg/100 g. The tannin content was similar among the components, except for oats flour, which had a significantly higher (*p* < 0.05) tannin content at 25.55 mg/100 g as compared to the others (Table 6). *Dabi* teff contained 92.8 mg/100 g of phytate and 14.07 mg/100 g of tannin, whereas field pea had a relatively higher phytate content at 158.89 mg/100 g.

**Table 6:** Anti-nutrient contents and phytate/minerals molar ratio of the individual components processed flours.

Individual flours	Ant-nutrients (mg/100 g)		Phytate/mineral molar ratio				
	Phytate	Tannin	Ph:Fe	Ph:Ca	Ph:Zn	Ph*Ca:Zn	
Dabi teff	92.8 ± 001^a^	14.07 ± 2.55^a^	0.091 ± 0.01^a^	0.046 ± 0.01^a^	4.711 ± 0.01^a^	14.556 ± 0.01^a^	
Roasted Barley	148.28 ± 0.37^b^	12.84 ± 0.59^a^	2.516 ± 0.01^b^	0.277 ± 0.01^b^	5.936 ± 0.01^b^	4.822 ± 0.01^b^	
Dehulled Oats	152.2 ± 0.1^c^	25.55 ± 1.27^b^	2.955 ± 0.01^c^	0.35 ± 0.03^c^	6.574 ± 0.01^c^	4.328 ± 0.01^c^	
Roasted Field pea	158.9 ± 0.1^d^	11.82 ± 0.01^a^	1.912 ± 0.01^d^	0.161 ± 0.01^d^	6.864 ± 0.01^d^	10.294 ± 0.01^d^	
Cooked Linseed	118.4 ± 0.28^e^	12.86 ± 0.54^a^	0.213 ± 0.01^e^	0.249 ± 0.01^be^	1.759 ± 0.01^e^	1.268 ± 0.01^e^	
Germinated Maize	80.6 ± 0.01^f^	13.01 ± 1.51^a^	6.106 ± 0.03^f^	0.31 ± 0.01^f^	5.088 ± 0.01^f^	2.007 ± 0.01^f^	
		Critical value	<1	<0.24	<15	<200	

Values are means ± Standard deviation of the triplicate determinations. Values in the same column followed by different superscripts are significantly different at *p* < 0.05.

### 3.3. Micronutrient contents of the formulated composite complementary flour

#### 3.3.1. Model fitting and model adequacy testing

The fitted models were found to be adequate and significant for most response variables based on the F-statistic (the ANOVA regressions outputs), the *p*-value, the coefficient of determinations *R*^2^, and the agreement between *R*^2^_adj_ and *R*^2^_pred_. In other words, the developed models were declared adequate for describing the effects of determinant variables (all the possible model terms) on the response variables. Table 4 shows that the linear model was adequately fitted for iron, zinc, Ph:Fe, Ph:Zn, and Ph*Ca:Zn contents at *p*-values of <0.05, whereas for calcium, the same linear model was suggested by the software, but it was not significant (*p* > 0.05). This shows that x_1_, x_4_, and x_6_ were the significant model terms for these variables with their corresponding *p*-values at 0.0001, 0.0164, and 0.1959 for iron, zinc, and calcium, respectively. This means that changes in iron, zinc, Ph:Fe, Ph:Zn, and Ph*Ca:Zn can be adequately described by the linear models (adequate predictive power) as a function of the component ratio variations in the formulations.

#### 3.3.2. Dietary mineral contents of the formulated composite complementary flours

The mean iron, calcium, zinc, magnesium, potassium, phosphorous, and sodium contents of the newly formulated composite complementary flours were determined to be 28.12 mg/100 g, 76.25 mg/100 g, and 2.47 mg/100 g, 120.57 mg/100 g, 443.99 mg/100 g, 293.25 mg/100 g, and 4.52 mg/100 g, respectively (Table 7). The new formulations incorporated with *dabi* teff flour had a significantly higher (*p* < 0.05) iron content (3.31–4.36 fold), iron density (3.25–4.27 fold), calcium content (1.49–1.58 fold), magnesium content (1.18–1.29 fold), and sodium content (1.54 fold), compared to the control flour. Zinc content was almost similar among the formulations. In this study, the mean iron content of the formulations ranged from 24.01 to 31.58 mg/100 g, whereas the mean calcium content ranged from 73.46 to 78.18 mg/100 g. The mean zinc content ranged from 2.33 to 2.61 mg/100 g (Table 7). The zinc content of the formulated flours was slightly lower than that of the control flour at 2.64 mg/100 g and that of the Cerifam^®^ faffa flour at 5 mg/100 g. The magnesium content of the new formulations ranged from 113.9 to 124.22 mg/100 g, while the sodium content ranged from 3.46 to 6.25 mg/100 g. There was no significant difference (*p* > 0.05) between the sodium content of the formulations and that of the control. The phosphorous and potassium contents of the newly formulated complementary flours ranged from 281.03 to 308.54 mg/100 g and 364.77 to 497.48 mg/100 g, respectively (Table 7).

**Table 7:** Dietary mineral contents of the formulated composite complementary fours.

Formulations	Micronutrient contents (mg/100 g)							
	Iron	Calcium	Zinc	Magnesium	Potassium	Phosphorous	Sodium	Iron density
F1	28.51 ± 0.17^a^	73.46 ± 0.85^a^	2.42 ± 0.02^a^	113.90 ± 2.01^a^	424.25 ± 10.25^a^	288.35 ± 4.31^a^	3.46 ± 0.66^a^	7.53
F2	27.86 ± 0.12^a^	76.48 ± 0.12^b^	2.58 ± 0.04^a^	121.52 ± 0.25^b^	482.48 ± 5.71^b^	297.87 ± 5.04^a^	4.64 ± 0.05^a^	7.31
F3	30.71 ± 0.23^bc^	76.63 ± 0.10^bc^	2.61 ± 0.02^b^	120.64 ± 0.72^bc^	488.25 ± 6.10^bc^	296.06 ± 5.26^a^	4.29 ± 0.04^a^	8.10
F4	24.01 ± 0.09^bd^	76.19 ± 0.11^bd^	2.50 ± 0.03^a^	120.54 ± 1.07^bd^	467.67 ± 2.91^bd^	308.54 ± 2.14^ab^	6.25 ± 0.05^b^	6.27
F5	30.33 ± 0.41^bc^	76.07 ± 0.18^bc^	2.42 ± 0.06^a^	122.72 ± 0.02^be^	364.77 ± 10.59^be^	291.03 ± 3.58^a^	3.97 ± 0.41^a^	7.90
F6	31.58 ± 0.37^bc^	78.18 ± 0.33^e^	2.45 ± 0.06^a^	118.22 ± 2.30^f^	427.37 ± 0.79^ac^	283.96 ± 6.37^ac^	4.60 ± 0.68^a^	8.25
F7	24.38 ± 0.09^bd^	75.25 ± 0.05^bf^	2.33 ± 0.08^ad^	122.18 ± 0.28^bg^	437.62 ± 3.74^ab^	292.29 ± 3.20^a^	4.04 ± 0.07^a^	6.33
F8	31.20 ± 0.12^bc^	77.57 ± 0.05^bg^	2.52 ± 0.06^a^	118.07 ± 0.08^h^	386.47 ± 7.42^cf^	281.03 ± 2.04^ac^	4.57 ± 0.03^a^	8.20
F9	24.51 ± 0.17^bd^	75.89 ± 0.05^bh^	2.39 ± 0.09^af^	121.07 ± 0.24^b^	497.48 ± 4.20^bg^	298.94 ± 4.40^a^	4.27 ± 0.04^a^	6.43
F10	25.68 ± 0.09^b^	76.22 ± 0.06^bd^	2.46 ± 0.08^a^	123.19 ± 0.11^be^	479.65 ± 9.48^bh^	300.15 ± 2.00^a^	5.69 ± 0.09^ad^	6.75
F11	30.58 ± 0.09^bc^	76.81 ± 0.11b^j^	2.52 ± 0.04^a^	124.22 ± 0.18^be^	427.87 ± 10.08^ab^	287.55 ± 4.61^a^	3.92 ± 0.02^c^	7.90
Mean	28.12	76.25	2.47	120.57	443.99	293.25	4.52	7.36
Control	7.25 ± 0.11^b^	49.45 ± 0.06^i^	2.64 ± 0.07^c^	96.45 ± 0.22^i^	398.67 ± 6.95^i^	282.65 ± 3.14^ad^	4.07 ± 0.04^a^	1.93
Optimal	32.21	77.52	2.59	118.64	439.33	287.99	4.44	8.47
FAO/WHO	16 mg/100 g	500	3.2	-	-	-	-	0.8–4
Cerifam^®^ Faffa	6	100^m^	5	-	-	-	-	-

Values are means ± Standard deviation of the triplicate determinations. Values in the same column followed by different superscripts are significantly different at *p* < 0.05.

#### 3.3.3. Anti-nutritional factors (phytate and tannin) of the formulated complementary flours

The mean values of phytate and tannin were determined to be 95.91 mg/100 g and 14.40 mg/100 g, and there observed a significant difference (*p* < 0.05) among the new composite formulations where their contents ranged from 85.2 to 104.12 mg/100 g for phytate and from 8.35 to 23.1 mg/100 g for tannin level (Table 8). The phytate content of the new formulations was significantly lower (*p* < 0.05) as compared to that of the control at 126.54 mg/100 g, and this could be attributed to the processing methods applied to the individual mixture components. However, tannin content was similar among most of the formulations and also to that of the control, which most of the formulations, which was determined at 12.42 mg/100 g.

**Table 8:** Anti-nutrient contents and phytate/minerals molar ratio of the formulated composite complementary flours.

Formulations	Mixture components ratio (%)						Anti-nutrient contents (mg/100 g)		Phytate/minerals molar ratio (*In-vitro* Bioavailability)			
	DTF	BAF	OF	FPF	LSF	GMF	Phytate	Tannin	Ph:Fe	Ph:Zn	Ph:Ca	Ph*Ca:Zn
F1	27.50	25	15	15.00	5	12.50	102.72 ± 1.19^a^	15.48 ± 2.29^a^	0.306 ± 0.01^a^	4.180 ± 0.03^a^	0.085 ± 0.01^a^	7.677 ± 0.01^a^
F2	26.123	25	15	23.877	5	5.00	96.8 ± 01^b^	13.01 ± 1.51^a^	0.295 ± 0.01^a^	3.695 ± 0.01^b^	0.077 ± 0.01^a^	7.065 ± 0.01^b^
F3	30.457	25	15	19.543	5	5.00	97.1 ± 01^b^	17.23 ± 3.1^a^	0.268 ± 0.01^a^	3.664 ± 0.01^bc^	0.077 ± 0.01^a^	7.019 ± 0.01^bc^
F4	20.00	25	15	29.988	5	5.012	96.94 ± 0.99^b^	18.06 ± 2.97^ab^	0.343 ± 0.01^ab^	3.819 ± 0.01^d^	0.077 ± 0.01^a^	7.274 ± 0.01^d^
F5	28.793	25	15	6.207	5	20.00	99.38 ± 2.08^b^	14.49 ± 2.31^a^	0.278 ± 0.01^a^	4.044 ± 0.01^e^	0.079 ± 0.01^a^	7.691 ± 0.01^a^
F6	35.00	25	15	11.884	5	8.116	86.27 ± 1.12^c^	10.64 ± 1.19^ac^	0.232 ± 0.01^be^	3.468 ± 0.01^f^	0.067 ± 0.01^a^	6.778 ± 0.15^e^
F7	21.705	25	15	13.295	5	20.00	97.61 ± 0.4^b^	11.44 ± 1.27^a^	0.340 ± 0.03^ae^	4.126 ± 0.01^g^	0.079 ± 0.01^a^	7.762 ± 0.01^a^
F8	34.385	25	15	6.476	5	14.139	85.2 ± 0.86^c^	23.1 ± 2.26^b^	0.232 ± 0.01^bc^	3.356 ± 0.01^h^	0.067 ± 0.01^a^	6.457 ± 0.01^f^
F9	20.00	25	15	19.540	5	15.460	97.3 ± 0.16^b^	8.35 ± 1.26^ac^	0.337 ± 0.01^ag^	4.009 ± 0.01^i^	0.078 ± 0.01^a^	7.607 ± 0.01^a^
F10	22.735	25	15	22.515	5	9.750	104.12 ± 0.13^a^	8.56 ± 0.85^ac^	0.344 ± 0.01^af^	4.168 ± 0.01^a^	0.083 ± 0.01^a^	7.943 ± 0.01^g^
F11	29.604	25	15	10.076	5	15.320	91.52 ± 0.21^d^	18.06 ± 1.44^ab^	0.254 ± 0.01^ad^	3.577 ± 0.01^j^	0.072 ± 0.01^a^	6.868 ± 0.01^e^
Average							95.91	14.40	0.29	3.83	0.076	7.29
Control	0	80				20	126.54 ± 0.82^e^	12.42 ± 1.09^a^	1.481 ± 0.03^e^	4.721 ± 0.01^k^	0.155 ± 0.01^b^	5.836 ± 0.01^i^
					Recommended values (Critical limits)				<1:1	<15:1	<0.24:1	<200:1

Values are means ± standard deviation of the triplicate determinations. Values in the same column followed by different superscripts are significantly different at *p* < 0.05. The corresponding component ratio for each formula code (F1- F11) is well described in Table 2.

#### 3.3.4. Phytate/minerals molar ratio of the formulated composite complementary flours (*in-vitro* mineral bioavailability)

The mean phytate/minerals (iron, zinc, and calcium) molar ratio of the newly formulated composite complementary flours is presented in Table 8. The phytate/iron molar ratio (Ph:Fe) ranged from 0.232 to 0.344 among the formulations, with a mean value of 0.29, which was far below the critical limit of 1 (Table 8). The phytate/calcium molar ratio (Ph:Ca) of the newly formulated complementary flours ranged from 0.067 to 0.085, with a mean value of 0.076, which was below the critical limit of 0.24:1; above this limit, calcium absorption can be impaired (17). The phytate/zinc molar ratio (Ph:Zn) ranged from 3.356 to 4.18 among the formulations, with a mean value of 3.83, while the Ph*Ca:Zn molar ratio (Ph*Ca:Zn) ranged from 6.457 to 7.943 among the formulations, with a mean value of 7.29, which was far below the critical limit of 200.

#### 3.3.5. Variable optimization and validation

**Figure 2:**
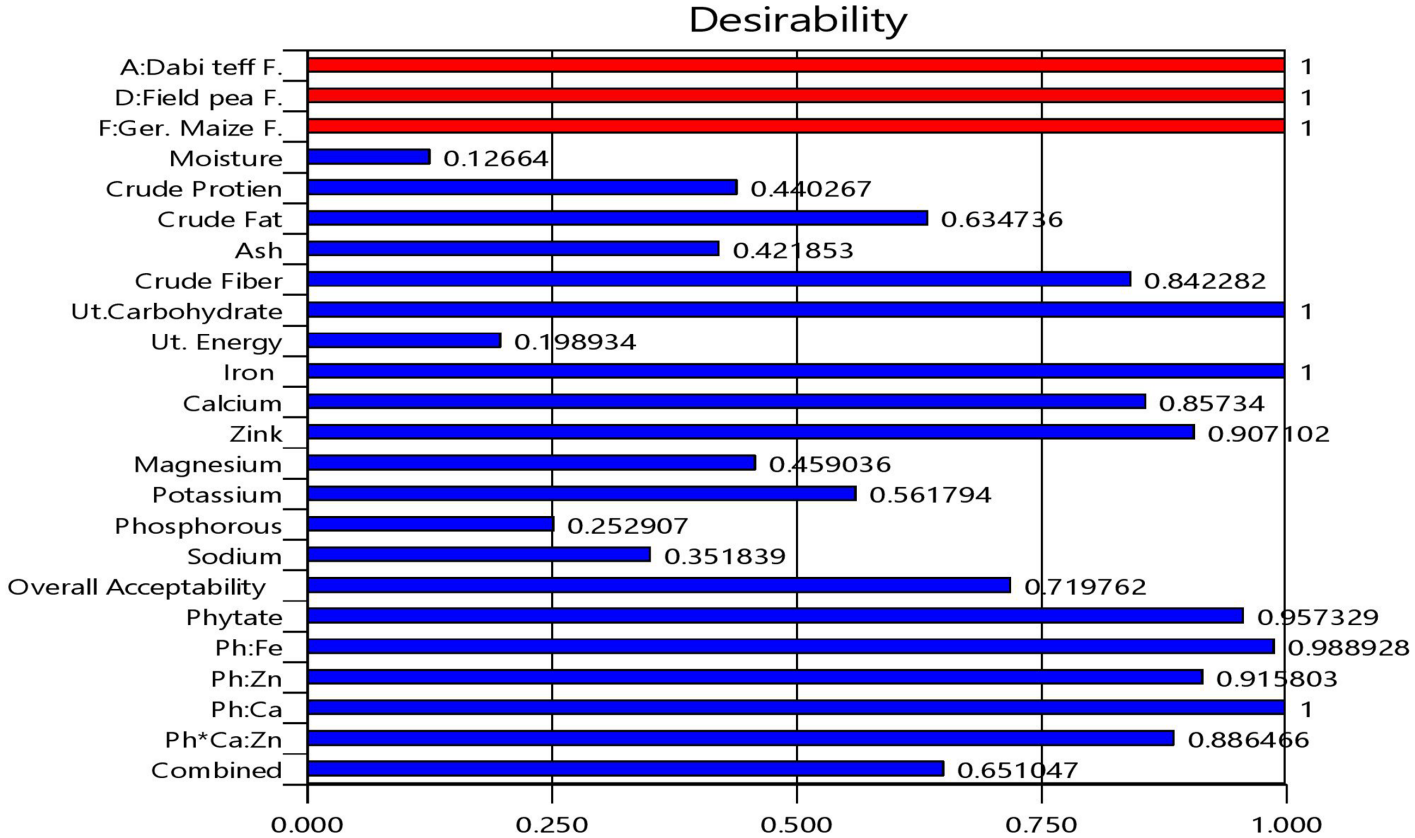
Bar graph showing the desirability functions of each response variable and the combined desirability function.

From the multi-response numerical optimization, the optimal formula (sweet spot) for the overall optimization was identified to be at 34.66% *dabi* teff flour, 25% barley, 15% oats, 15.34% field pea, 5% linseed, and 5% germinated maize flours, with the predicted optimal value for the dietary minerals at 32.21 mg/100 g for iron, 77.51 mg/100 g for calcium, 2.59 mg/100 g for zinc, 118.64 mg/100 g for magnesium, 439.33 mg/100 g for potassium, 287.99 mg/100 g for phosphorous, 4.44 mg/100 g for sodium, 86.01 mg/100 g for phytate, 0.233 for the Ph:Fe molar ratio, 3.43 for the Ph:Zn molar ratio, 0.067 for the Ph:Ca molar ratio, and finally, 6.63 for the Ph*Ca:Zn molar ratio (Table 5), where the maximum selected combined desirability function was 0.651 (Figure 2). The results obtained at the optimum conditions showed significantly higher iron (4.44 fold), calcium (1.57 fold), and magnesium (1.23 fold) than that of the control; thus, the optimized novel composite complementary flour demonstrated superiority (*p* < 0.05) in terms of minerals, specifically in terms of the iron content, over the traditional complementary flour (Table 7).

**Figure 3:**
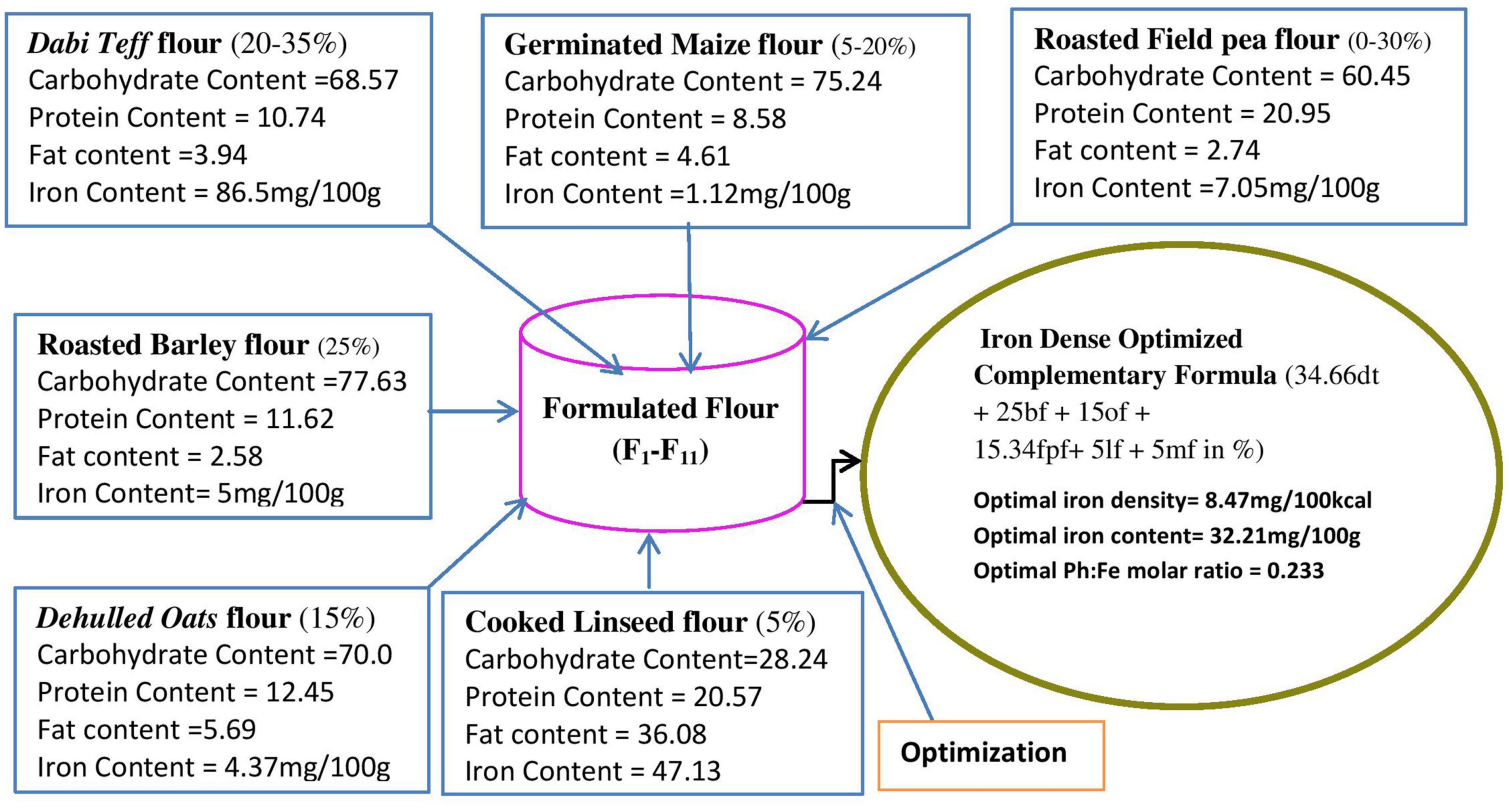
Material balance design showing individual flour ranges and their nutrient contents and the optimal condition identified. dt, dabi teff; bf, barley; of, oats; fp, field pea; If, linseed; mf, maize flours.

Figure 3 shows the material balance design of the defined ranges of the components, their nutrient contents, and the optimal condition identified.

**Figure 4:**
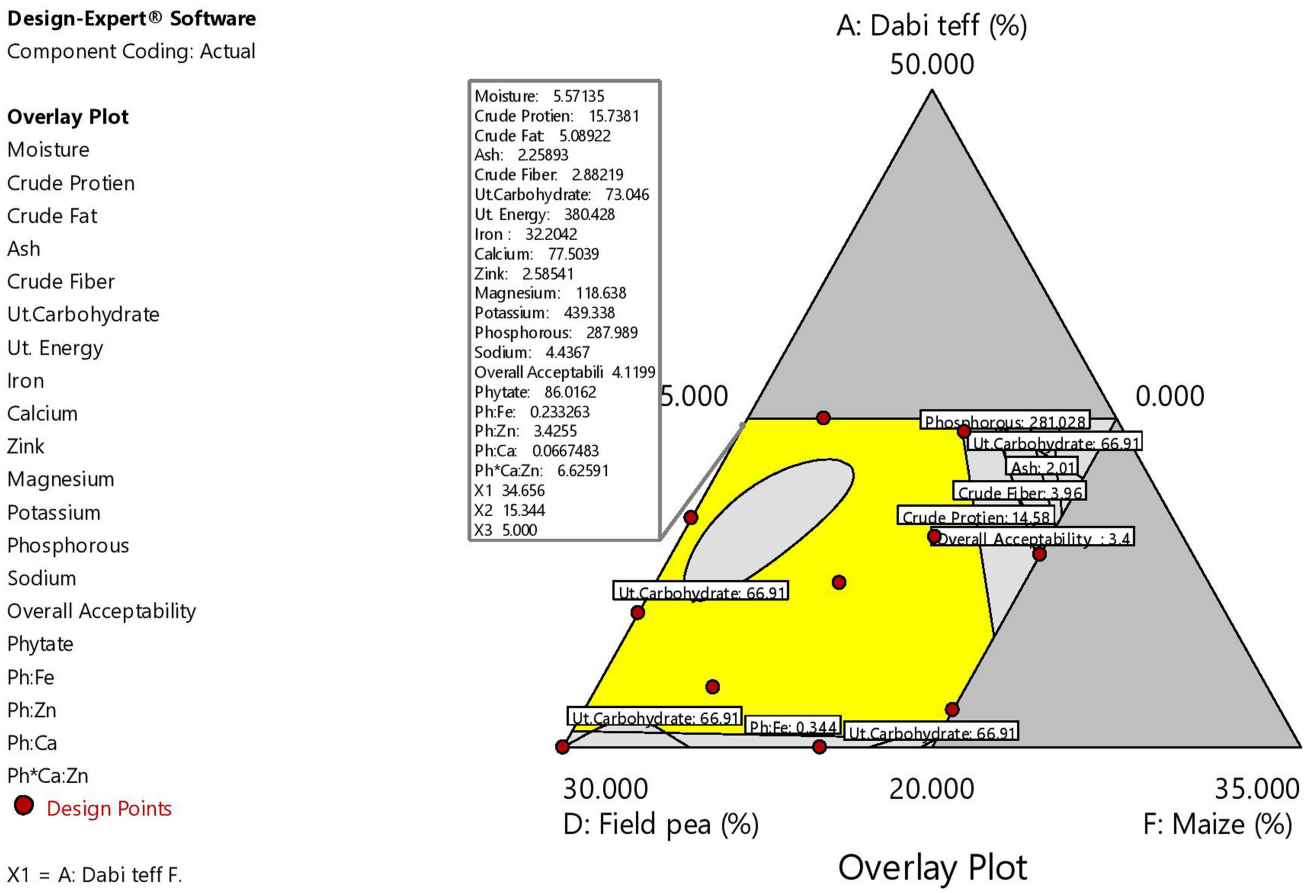
Graphical overlay contour plot for the overall graphical optimization.

To validate the optimal condition, the ratio of each component at the optimized formula was blended, and laboratory analysis of the micronutrient compositions of the blend was conducted. The validation analysis results were 31.76 mg/100 g for iron, 76.88 mg/100 g for calcium, 2.53 mg/100 g for zinc, 119.61 mg/100 g for magnesium, 440.66 mg/100 g for potassium, 291.48 mg/100 g for phosphorous, 4.48 mg/100 g for sodium, 90.93 mg/100 g for phytate, 0.236 for Ph:Fe, 3.52 for Ph:Zn, 0.074 for Ph:Ca, and 7.22 for Ph*Ca:Zn. These values and the predicted response values at the optimal condition were significantly higher at a value of p of <0.05 than those of the control flour, except for zinc and phosphorous contents (Table 9), which showed the superiority of the optimized composite novel complementary flour over the control flour. Furthermore, the predicted response values derived from the model equations at the optimal and experimental (validation) values obtained under laboratory analysis are presented in Table 10, which shows that there was no significant difference (*p* > 0.05) between the predicted optimal response values and the validation (laboratory analysis) values, except for potassium (slight difference). This showed that there was a good agreement between the predicted optimal response values at optimal conditions and the validation values, thereby confirming the validity and adequacy (accuracy) of the model equations for predicting the optimal response values. The optimization process was verified using the graphical optimization technique (Figure 4), where the overlay counter plot was generated for the most important variables to identify the optimum regions. Any design point that falls within the yellow region (within the very conservative silver-gray region) in the overlay plot represents an optimal combination of *dabi* teff flour, field pea, and germinated maize flour in combination with the other constant components that can bear the optimal micronutrient contents with the phytate/minerals molar ratio reduced to the minimum within 95% confidence interval (CI).

**Table 9:** Experimental validation of optimal condition and comparison with the control flour.

Response variables	Optimized value	Validation value	Contro value
Iron (mg/100 g)	32.21^a^	31.76^a^	7.25^b^
Calcium (mg/100 g)	77.51^a^	76.88^a^	49.45^b^
Zinc (mg/100 g)	2.59^a^	2.53^a^	2.64^a^
Magnesium (mg/100 g)	118.64^a^	119.61^a^	96.45^b^
Potassium (mg/100 g)	439.33^a^	440.66^a^	398.67^b^
Phosphorous (mg/100 g)	287.99^a^	291.48^a^	282.65^a^
Sodium (mg/100 g)	4.44^a^	4.48^a^	4.07^b^
Phytate (mg/100 g)	86.01^a^	90.93^a^	126.54^b^
Ph:Fe	0.233^a^	0.236^a^	1.48^b^
Ph:Zn	3.43^a^	3.52^a^	4.72^b^
Ph:Ca	0.067^a^	0.074^a^	0.16^b^
Ph*Ca:Zn	6.63^a^	7.22^a^	5.84^b^

Values are means ± Standard deviation of the triplicate determinations. Values in the same row followed by the same superscripts are not significantly different at *p* > 0.05.

**Table 10:** Predicted optimal response values and experimental (validation) values for verification.

Response variables	Optimized value	Validation value
Iron (mg/100 g)	32.21^a^	31.76^a^
Calcium (mg/100 g)	77.51^a^	76.88^a^
Zinc (mg/100 g)	2.59^a^	2.53^a^
Magnesium (mg/100 g)	118.64^a^	119.61^a^
Potassium (mg/100 g)	439.33^a^	440.66^b^
Phosphorous (mg/100 g)	287.99^a^	291.48^a^
Sodium (mg/100 g)	4.44^a^	4.48^a^
Phytate (mg/100 g)	86.01^a^	90.93^a^
Ph:Fe	0.233^a^	0.236^a^
Ph:Zn	3.43^a^	3.52^a^
Ph:Ca	0.067^a^	0.074^a^
Ph*Ca:Zn	6.63^a^	7.22^a^

Values are means ± Standard deviation of the triplicate determinations. Values in the same row followed by the same superscripts are not significantly different at *p* > 0.05.

#### 3.3.6. Micronutrient densities of the optimized complementary flour

The micronutrient densities of the optimized, the control, the Cerifam^®^ faffa flour, and the PAHO/WHO (11) recommendations for complementary foods for average breast-fed children are presented in Table 11. The iron density of the optimized complementary flour at 8.47 mg/100 kcal was compared to the recommendation, and it accounted for high, moderate, and low bioavailability. The recommended values for 6–8, 9–11, and 12–23 month-old children are 2.5 mg/100 kcal, 1.5 mg/100 kcal, and 0.5 mg/100 kcal when accounting for high bioavailability, 4.0 mg/100 kcal, 2.4 mg/100 kcal, and 0.8 mg/100 kcal when accounting for moderate bioavailability, and 7.7 mg/100 kcal, 4.6 mg/100 kcal, and 1.6 mg/100 kcal when accounting for low bioavailability, respectively (11, 38, 39). The determined zinc density of the new formulations was at 0.68 mg/100 kcal, while the calcium density was at 20.37 mg/100 kcal.

**Table 11:** Micronutrient densities of the composite complementary flours and Codex standards.

Computed responses (mg/100 kcal)	Optimal flour	Control flour	PAHO/WHO	Cerifam^®^
Iron density	8.47	1.93	0.8–4	1.52
Calcium density	20.37	13.20	>80	25.25
Zinc density	0.68	0.70	0.6–1.6	1.26
Magnesium density	31.19	25.74	-	-
Phosphorous density	75.70	75.43	-	-

## 4. Discussion

Minerals are vital nutrients for life. They are essential for healthy brain, bone, and body development and are also growth regulators in children (1). In the present study, the iron content of *dabi* teff was determined to be 86.5 mg/100 g, which was much higher than that of commonly recommended animal source foods for iron, such as liver (17.9 mg/100 g edible portion) and red beef meat (3.79 mg/100 g) (40). However, the problem with plant-source iron is its non-heme form, limiting its absorption. This problem can be resolved by suggesting the consumption of vitamin C-rich foods, such as citrus fruits. When compared to the FAO/WHO (25) recommendation, *dabi* teff was shown to have 5.4 times higher iron content than the requirement from complementary food; additionally, it had an iron density of 24.53 mg/100 kcal, given its energy value of 352.70 kcal/100 g (41), making the crop a super iron bank. Contrary to the present findings, a lower level of iron content (38 mg/100 g) was reported by Mezgebo et al. (42) for the popular red teff grown in Ethiopia. Similarly, Daba (28) reported lower iron content of the red-teff (Dz-01-1681) variety at 20.6 mg/100 g. The high iron level of *dabi* teff determined in this study magnified the importance of incorporating the crop into other pre-processed local food crops to develop iron-dense optimized novel composite complementary products.

In agreement with the present findings, Woldemariam et al. (43) reported the calcium content of the popular red teff at 102.96 mg/100 g. Even though it may not be reasonable to compare the nutrient contents of cereals and legumes because of their different natural physiognomies, in the present study, we compared them simply because they were our components. We also compared them to justify that cereals and legumes need to be mixed to nutritionally complement each other to develop wholesome and nutritionally complete end products. Aligned with this, the nutrient contents of *dabi* teff, specifically zinc and magnesium, were incomparably higher (in some cases similar) than those of other cereals, such as sorghum (44), although they were lower than some of our current components.

Compared to the critical limits, the phytate and tannin contents of all the individual components were below the maximum tolerable limit at 200 mg/100 g for phytate and 560 mg/100 g for tannin (17, 45). These two anti-nutrients are of concern in limiting the absorption and bioavailability of nutrients from cereals and legumes, but currently, we do not foresee any significant problems because of their determined low levels in all our components. Specifically, *dabi* teff flour, which contained the highest iron and calcium contents (Table 1), had the lowest phytate and tannin contents, and the phytate/iron molar ratio was calculated to be 0.091 (Table 6), which was far below the critical limit (<1) (17). This demonstrates the good proxy indicator of iron absorption and bioavailability from *dabi* teff-field pea-based complementary food products. These lower anti-nutrient values of *dabi* teff and others add to the justification of our components as the best candidates in the formulations.

Iron is a vital nutrient necessary for building and maintaining healthy red blood cells and for making hemoglobin (1). When compared to the FAO/WHO (25) guidelines for the nutrient content of formulated complementary foods, the mean value of the new formulations had a significantly higher (*p* < 0.05) mean iron content (1.76 fold), where the recommended value is at 16 mg/100 g, whereas none of the formulations met the recommendations for calcium and zinc at 500 mg/day and 3.2 mg/100 g, respectively, which may call for searching for other calcium- and zinc-rich food sources.

Compared to the present findings, Mezgabo et al. (42) reported a wider range of iron content (9.38–34.86 mg/100 g) for a complementary porridge formulation from red teff, malted soybean flour, and papaya fruit powder, but it is in reasonable agreement with our results. Contrarily, the present finding was higher than that of the report by Alaunyte et al. (46), who reported that by supplementing wheat bread with 30% teff flour, the iron content of the bread was more than doubled, whereas, in our formulations, it was more than tripled (3.31–4.36 fold) compared to control, with *dabi* teff ranging from 20 to 35%. Similarly, when compared to Cerifam^®^ faffa flour, the new formulations had a significantly higher (p < 0.05) iron content (4.0–5.26 fold).

Contrary to the present finding, Mezgebo et al. (42) reported a higher calcium content at 293.57 mg/100 g for a complementary porridge formulation from red teff, malted soybean flour, and papaya fruit powder, and the report associated the high calcium content of the formulations with the higher calcium content in soybean flour. Cerifam^®^ faffa flour also had a significantly higher (*p* < 0.05) calcium content than our new formulations, which could be attributed to the milk powder contained in the product.

Furthermore, contrary to the present findings, Mezgebo et al. (42) reported a higher zinc content, which ranged from 4.05 to 5.58 mg/100 g for complementary porridge formulation from red teff, malted soybean flour, and papaya fruit powder. Magnesium is a cofactor that aids in protein synthesis in the human body, and its deficiency in children leads to stunted growth (1). In the current study, we observed a high mean magnesium content of the newly developed complementary flour at 120.57 mg/100 g. The literature supports that cereals, legumes, and oilseeds contain a good amount of magnesium, and the presence of these components in the current formulation was responsible for the higher magnesium content.

Sodium is an essential mineral mostly responsible for the maintenance of extra-cellular fluid and normal blood pressure, along with other several physiological functions. The moderate use of salt in some foods is appropriate, and it is neither safe nor reasonable to avoid salt from infants’ and younger children’s diets (47). In this study, the sodium content of the new formulations was far below the recommended dietary allowance (RDA) for infants (370 mg/day) and young children (1,000 mg/day) (47). This very low sodium content might call for the addition of a small amount of iodized salt during complementary porridge preparation. The new formulations had a vital amount of phosphorous at 293.25 mg/100 g; the principal role of phosphorus in the human body is that it combines with calcium to make bones and teeth, and it is used to release energy from carbohydrates and fat during metabolism (ATP production).

The present findings showed that phytate and tannin contents in the formulated flours were below the maximum tolerable level at 200 mg/100 g (17, 45) for phytate and at (560 mg/100 g) for tannin, which indicates that both anti-nutrients might not possibly inhibit the digestion, absorption, and bioavailability of minerals and protein from the formulated complementary food.

The anti-nutrients/minerals molar ratio has more nutritional implications than the anti-nutrient contents of food. That is, the degree of interaction between the minerals and the anti-nutrients to form insoluble complexes determines the absorption inhibitory effect, thus influencing the bioavailability of the minerals under study. In the human body, this effect can be predicted by calculating the molar ratios of the anti-nutrients to minerals (30), which shows the proxy indicator of minerals’ bioavailability. The mean calculated value of the Ph:Fe molar ratio for the formulations at 0.29 was far below the critical value at 1:1 ratio, where a value greater than this has a negative effect on iron absorption; and it was even lower than 0.4:1, which is the preferable Ph:Fe molar ratio (45, 48). The critical limit for (Ph:Zn) is 15:1 (Table 8), where a value greater than this limit is associated with low estimated bioavailability, and 5–15 and below 5 Ph:Zn molar ratios are associated with moderate and high estimated zinc bioavailability, respectively, with zinc absorption corresponding to 15, 30, and 50%, respectively (48). The calculated value in the current study fell in the third category (Ph:Zn below 5), showing higher estimated bioavailability with an expected 50% zinc absorption rate.

In infants and young children, zinc absorption is not directly related to dietary phytate intake but indirectly related to the dietary calcium content. Higher calcium content in foods induces the dietary phytate to reduce zinc absorption by potentiating the negative effect of phytate through the formation of insoluble phytate-calcium-zinc complexes, and calcium itself does not impair zinc absorption, regardless of whether the phytate is high or low (49). Thus, the presence of higher calcium content in diets has been suggested to be a better predictor of zinc absorption, and the Ph*Ca:Zn molar ratio is the preferred indicator of zinc absorption from foods rather than the Ph:Zn molar ratio, where a Ph*Ca:Zn molar ratio greater than the critical limit (200,1) may lead to reduced/poor zinc bioavailability (30). In the present findings, the Ph*Ca:Zn molar ratio was incomparably lower than the critical limit, which showed the reduced likelihood of zinc malabsorption expected from the complementary diet. In reality, not calcium from a conventional diet but calcium supplementation is expected to impede zinc absorption (45, 48). This was confirmed by a previous report by Hemalatha et al. (50), which stated that zinc absorption and bioavailability are unlikely to be impaired by the calcium contents of foods prepared from cereals and pulses. The probable reason for these low phytate/mineral molar ratios may be the effect of soaking, germination, roasting, and dehulling processes applied on the components used in our study. Gibson et al. (51) reported that the molar ratios of phytate/mineral were improved (lowered) by practicing processing techniques, such as roasting, germination, and fermentation, or by enriching complementary foods with animal-source foods.

Optimized composite complementary flour that can meet iron density requirements with reduced phytate/minerals molar ratios can be obtained from nutritious cereal–legume blends to develop a super-quality complex child food. Child nutrition emphasizes the importance of quality protein, omega-3 polyunsaturated fatty acid, and adequate iron during complementary feeding (11, 25), where our new optimized product certainly contains a substantial amount of these nutrients owing to the presence of field pea, linseed, and *dabi* teff flours in the formulations.

The present computed phytate/minerals molar ratios of the optimized complementary flour were far below the critical limits at <1, <0.24, <15, and < 200 for Ph:Fe, Ph:Ca, Ph:Zn, and Ph*Ca:Zn, respectively, as set by the FAO/WHO (25), indicating the unlikely absorption inhibition effect of phytate on the minerals.

Regarding the maximum combined desirability function, Talabi et al. (52) reported a desirability function similar to that of the present study, at 0.65, during the optimization of the complementary meal from a mix of yellow maize, sorghum, millet, soybean, groundnut, crayfish, and fish. Additionally, Keyata et al. (53) reported a similar desirability function of 0.625 for the overall optimization of their complementary food from fro sorghum, soybean, karkade, and premix.

When compared to the FAO/WHO (25) guidelines for the nutrient content requirements of formulated complementary flour, the newly optimized novel composite complementary flour adequately met the recommended standard for iron at 16 mg/100 g. This shows that the optimized complementary flour could demonstrate two times higher iron content than the recommended value, demonstrating the superiority of our optimized complementary flour over the recommended standard and the control flour. It may not be surprising that the optimized complementary flour had two times higher iron content (32.21 mg/100 g) than the recommended value, at 16 mg/100 g, because *dabi* teff flour showed an extraordinarily high iron content at 86.5 mg/100 g (Table 1).

Zinc met the recommended value (3.2 mg/100 g) at a marginal of 80.94%, which might be taken as a considerable amount, whereas calcium did not meet the recommendation at 500 mg/100 g.

The ‘novelty’ of the newly optimized novel composite complementary flour developed could be accredited to the incorporation of the *dabi* teff flour with its high iron content (86.5 mg/100 g) and good bioavailability (Ph:Fe molar ratio far below the critical limit), and the results demonstrated substantial evidence for the social beliefs regarding this typical farmer-variety teff. In addition, linseed flour, which is a leading source of α-Linolenic acid, and omega-3 polyunsaturated fatty acid would make the product super.

According to the PAHO/WHO (11) complementary feeding guidelines, the iron density of cereal–legume-based complementary foods for infants and young children (6–23 months) should range from 0.8 to 4.0 mg/100 kcal when accounting for moderate iron bioavailability, and the iron density of our current optimized complementary flour was computed to be 8.47 mg/100 kcal, given the 380.43 kcal/100 g energy value of our optimized complementary flour (41), which was 2.12–10.59 times higher than the recommended value when accounting for moderate bioavailability and 4.39 and 5.57 times higher than those of control and the Cerifam^®^ faffa flour when accounting for low bioavailability, which could be accredited to the higher iron content of *dabi* teff flour.

This finding showed that the newly optimized complementary flour could even meet the recommendation when accounting for the low bioavailability of iron density at 7.7 mg/100 kcal. Hence, the newly optimized complementary flour could meet the iron density requirements for infants and young children aged 6–23 months, indicating that children can meet their daily iron demand if properly fed. The iron density reported in the present finding was higher than the iron density of complementary blends of teff, soya bean, and orange-fleshed sweet potato, which ranged from 2.42 to 5.19 mg/100 kcal, as reported by Tenagashaw et al. (54). Iron in plant foods exists in the non-heme form, and it may also bind with anti-nutrients found in cereal–legume foods, where its absorption can be impaired (45). However, in our case, the pre-processing technique applied might have reduced the effect of the anti-nutrients, as a result of which the Ph:Fe molar ratio was far below the critical limit.

The PAHO/WHO (11) recommends zinc density at 1.6 mg/100 kcal, 1.1 mg/100 g, and 0.6 mg/100 g for children aged 6–8 months, 9–11, and 12–23 months from complementary foods when accounting for lower bioavailability (39), and the current determined zinc density was at 0.68 mg/100 kcal, showing fulfillment for 12–23 months old children while at the marginal level (61.82%) when accounting for the requirements for 9–11 months old children and below the recommendation for 6–8 months old children. The zinc density reported in the present finding was higher than the zinc density of complementary food at 0.4 mg/100 kcal, as reported by Ayele et al. (55), while it was lower than that reported by Tenagashaw et al. (54), with a zinc density range of 1.41 to 1.49 mg/100 kcal.

In the current study, the calcium density could only meet 25.46% of the required calcium density in formulated complementary foods for infants and young children, as set by the Codex standard (>80 mg/100 kcal) (25). The density reported in the present finding was slightly lower than that reported by Ayele et al. (55), where the calcium density was at 24.1 mg/100 kcal. However, it was far lower than that reported by Tenagashaw et al. (54), with a calcium density range of 60.68 to 67.84 mg/100 kcal from complementary blends of teff, soya bean, and orange-fleshed sweet potato, which could be attributed to the variety of soya bean and the orange-fleshed sweet potato used. Among the examined micronutrients, calcium density was found to be lower than the required amount, and it has frequently been reported to be low in cereal-based complementary foods (56, 57).

The current study could have practical applications in the industrial production of iron-dense baby food and in the preparation of *dabi* teff-field pea-based iron-enriched homemade complementary foods targeting infants and young children, school-going children, pregnant and lactating women, as well as women of reproductive age group starting from the menarche stage. It could be utilized to meet the optimal levels of bioavailable iron-rich food products. The higher iron content and iron density with improved *in vitro* bioavailability of the newly developed *dabi* teff-field pea-based product could increase the hemoglobin level of the blood, which allows for more oxygen to be transmitted and aids in higher resistance and increased endurance during athletics performance. For example, recent studies have shown that athletic runners require 30–70% more iron due to losses from foot strike hemolysis and gastrointestinal blood loss (58). Thus, the consumption of the newly developed product (which can be scaled up at the industrial level) could provide this higher iron requirement among athletic runners, thereby being a magnificent implication in sports nutrition.

## 5. Future recommendations

Further studies will be required to substantiate the major findings of this study. These studies might include examining the functional properties of the flour, adding animal-source foods to match the lower levels of calcium and zinc, exploring combined and varying processing conditions on nutritional and anti-nutritional contents, and conducting randomized controlled feeding trials. These trials can be combined with nutrition education through behavioral change communication (BCC) aimed at mothers/caretakers to determine the efficacy of the newly developed *dabi* teff-field pea-based optimized novel complementary flour in improving iron status among infants and young children. It is further recommended that *dabi* teff be integrated into the Ethiopian agricultural research program for its extensive production, industrial utilization, and home consumption.

## 6. Conclusion

Micronutrient malnutrition, especially iron deficiency anemia, is the most prevalent in children under two years than at any other time in the life cycle. In the present study, the incorporation of *dabi* teff into other pre-processed local food crops provided an iron-dense optimized novel complementary flour that met the FAO/WHO guideline recommendations and exhibited improved *in-vitro* iron bioavailability over the traditional and the Cerifam^®^ faffa flour. Each of the components used contributed important limiting micronutrients, such as iron, calcium, and zinc, for developing a micronutrient-enriched optimized product, where the *dabi* teff grain, a typical farmer-variety teff was found to contain 86.5 mg/100 g of iron, with an iron density of 24.53 mg/100 kcal, making the crop a super iron bank. The optimized flour had an iron density of 8.47 mg/100 kcal. The results showed that the iron content of the optimized flour was two times higher and the iron density was 2.12–10.59 times higher than the recommended value from complementary foods when accounting for moderate bioavailability. It was even shown to meet the recommendation at low bioavailability. The findings further showed that the optimized novel complementary flour contained 4.44 times higher iron content, 1.57 times higher calcium content, and 1.23 times higher magnesium content, as compared to the control. The optimized flour was also shown to have 5.57 times higher iron density as compared to the Cerifam^®^ faffa flour, and the phytate/iron molar ratio was very low, showing higher bioavailable iron. Thus, in Ethiopia, where the consumption of animal-source foods is low and where nearly all iron requirements (>97%) should come from complementary foods after 6 months, the promotion of local forgotten food crops, such as *dabi* teff, in the formulation of complementary foods is required. *Dabi* teff is a good dietary source of iron from cereals that can serve as an inexpensive and sustainable way of meeting children’s iron demand for their optimal growth and development, and it can be used to aid sustainable food-based intervention strategies to combat iron deficiency anemia.

## Data availability statement

The original contributions presented in the study are included in the article/supplementary material, further inquiries can be directed to the corresponding author.

## Author contributions

DCT: conceptualization, designing, formal analysis, interpretations, methodology, experimentation, software, writing original draft, and writing–review and editing. TB, DT, and KA: conceptualization, designing, methodology, analysis, interpretations, critical revision of the manuscript, and writing–review and editing. All authors contributed to the article and approved the submitted version.
